# The Interface Between Wheat and the Wheat Curl Mite, *Aceria tosichella*, the Primary Vector of Globally Important Viral Diseases

**DOI:** 10.3389/fpls.2018.01098

**Published:** 2018-07-27

**Authors:** Anna Skoracka, Brian G. Rector, Gary L. Hein

**Affiliations:** ^1^Population Ecology Lab, Faculty of Biology, Adam Mickiewicz University, Poznań, Poland; ^2^Great Basin Rangelands Research Unit, United States Department of Agriculture – Agricultural Research Service, Reno, NV, United States; ^3^Department of Entomology, University of Nebraska-Lincoln, Lincoln, NE, United States

**Keywords:** cereals, eriophyoid mites, pathogen vector, plant viruses, phytophagous mites

## Abstract

Wheat production and sustainability are steadily threatened by pests and pathogens in both wealthy and developing countries. This review is focused on the wheat curl mite (WCM), *Aceria tosichella*, and its relationship with wheat. WCM is a major pest of wheat and other cereals and a vector of at least four damaging plant viruses (*Wheat streak mosaic virus*, *High plains wheat mosaic virus*, *Brome streak mosaic virus*, and *Triticum mosaic virus*). The WCM–virus pathosystem causes considerable yield losses worldwide and its severity increases significantly when mixed-virus infections occur. Chemical control strategies are largely ineffective because WCM occupies secluded niches on the plant, e.g., leaf sheaths or curled leaves in the whorl. The challenge of effectively managing this pest–virus complex is exacerbated by the existence of divergent WCM lineages that differ in host-colonization and virus-transmission abilities. We highlight research progress in mite ecology and virus epidemiology that affect management and development of cereal cultivars with WCM- and virus-resistance genes. We also address the challenge of avoiding both agronomically deleterious side effects and selection for field populations of WCM that can overcome these resistance genes. This report integrates the current state of knowledge of WCM–virus-plant interactions and addresses knowledge gaps regarding the mechanisms driving WCM infestation, viral epidemics, and plant responses. We discuss the potential application of molecular methods (e.g., transcriptomics, epigenetics, and whole-genome sequencing) to understand the chemical and cellular interface between the wheat plant and WCM–virus complexes.

## Introduction

Wheat, *Triticum aestivum* L., is the most abundant source of calories and protein in the human diet (Braun et al., 2010; Arzani and Ashraf, 2017). It is grown annually on 215 million acres, an area larger than for any other crop, and remains the most traded on world markets and the most important crop in the 21st Century (Curtis and Halford, 2014).

**FIGURE 1:**
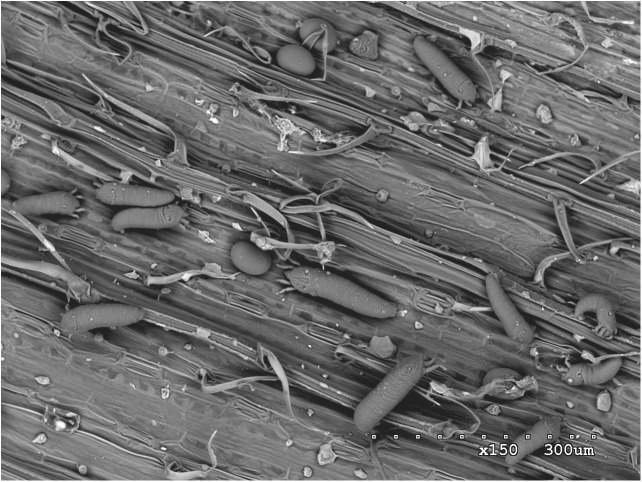
Scanning electron microscopy (SEM) image of wheat curl mite (*Aceria tosichella*) specimens on a wheat leaf.

**FIGURE 2:**
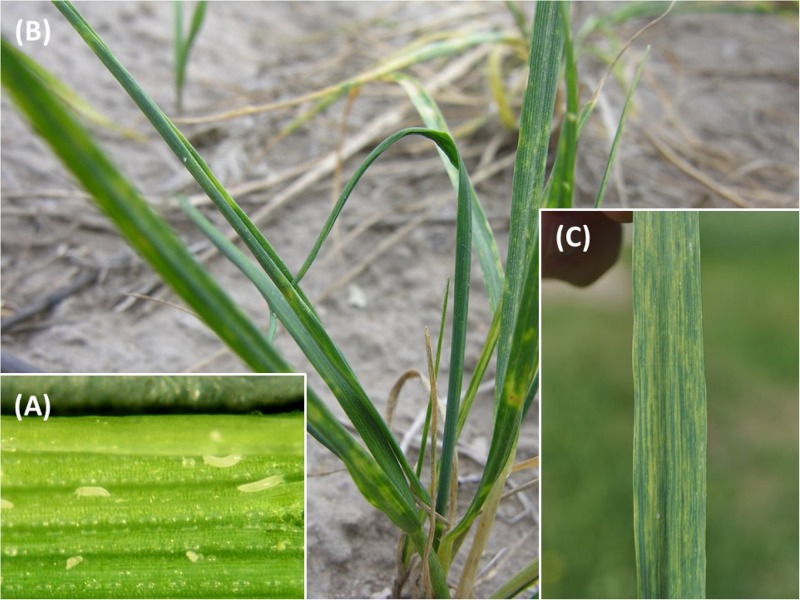
Wheat curl mite (WCM) and WSMV symptoms: **(A)** specimens of WCM on a wheat leaf; **(B)** leaf curls caused by WCM; and **(C)** WSMV symptoms on wheat leaf.

However, wheat production is affected by a number of pests, including insects, fungi, nematodes, and mites, that can severely reduce yields and lead to crop failures. One of the most important global pests of wheat, occurring in North and South America, Europe, Asia, and Oceania, is the wheat curl mite (WCM), *Aceria tosichella* Keifer (**Figures 1**, **2A**) which belongs to the superfamily Eriophyoidea (Navia et al., 2013). WCM is minute (about 0.2 mm long) and occupies sheltered niches on the plant, such as leaf sheaths and rolled and curled leaves, making its detection difficult, and limiting its exposure to acaricides (Navia et al., 2013). Moreover, its reproduction by arrhenotokous parthenogenesis (Miller et al., 2012), short developmental time, and high thermal tolerance (Kuczyński et al., 2016) enable great colonization potential.

The greatest economic impact of WCM results from its ability to transmit at least four damaging plant viruses to several different cereal crops. In this review we integrate the current state of knowledge of WCM–virus-plant interactions and address knowledge gaps regarding the mechanisms driving WCM infestation, viral epidemics, and plant responses.

## What Curl Mite Feeding and Virus Transmission

Almost 90 grass species worldwide have been reported as host plants for WCM including cereals such as wheat, oats, barley, pearl millet, corn, and rye, as well as other cultivated (pasture) and uncultivated grasses (Navia et al., 2013). WCM has very short chelicerae (ca. 0.02 mm) and can feed only on leaf epidermal tissues. On wheat they colonize the plant by feeding within the whorl of a developing leaf on thin-walled epidermal tissue known as bulliform cells. Feeding on these cells by mites prevents leaves from unfurling causing leaf curling (**Figure 2B**) that promotes a humid environment generally preferred by WCM. WCM feeding also reduces photosynthetic capacity (Royalty and Perring, 1996).

The WCM has been shown to be the only transmitter of four distinct viruses to wheat and numerous other grass hosts (Stenger et al., 2016). These viruses occur across two virus families and three virus genera. Slykhuis (1955) first identified WCM as the vector of *Wheat streak mosaic virus* (family *Potyviridae*/genus *Tritimovirus*; acronym WSMV). The mite was also shown to transmit *High plains wheat mosaic virus* (*Fimoviridae*/*Emaravirus*; HPWMoV) (Seifers et al., 1997). Transmission of *Brome streak mosaic virus* (*Potyviridae*/*Tritimovirus*; BrSMV) by WCM was verified by Stephan et al. (2008). Most recently, Seifers et al. (2009) identified the WCM as the vector of *Triticum mosaic virus* (*Potyviridae*/*Poacevirus*; TriMV).

Of these viruses, WSMV is the most widely distributed and studied and it has been identified from every major wheat growing region around the world (Navia et al., 2013). The greatest and most consistent impact from WSMV occurs across the Great Plains of North America with more sporadic impact in other regions. BrSMV has only been found in Europe and no economic impact from the virus has been reported (Stephan et al., 2008).

*Wheat streak mosaic virus* infection of wheat results in a light and dark green mosaic pattern on the youngest emerged leaves (**Figure 2C**; Wegulo et al., 2008). As the plant adds new leaves, the newest leaves will first show these subtle mosaic symptoms while older leaves will become progressively more yellow. The tight curling at the leaf edge resulting from mite feeding is often apparent. The severity of symptoms and subsequent yield impact from virus infection in wheat depends on the plant stage at first infection (Hunger et al., 1992; Wosula et al., 2018). Plants infected prior to or during tillering will eventually become severely stunted, discolored, and take on a very prostrate growth pattern. These severe symptoms indicate that extreme yield loss will occur.

In the North American Great Plains co-infection of the viruses is common (Burrows et al., 2009; Byamukama et al., 2013, 2016) and may result in more spotted appearance on leaves but distinguishing symptoms of co-infections is not possible. Co-infection of WSMV and TriMV have been shown to increase the severity of symptoms and yield impacts (Tatineni et al., 2010; Byamukama et al., 2012, 2014). HPWMoV is not manually transmissible and this has limited study of this virus both independently and in combination with other viruses (Tatineni et al., 2014; Stenger et al., 2016).

## WCM Diversity and its Implications

Understanding the relationships between WCM, viruses, and their hosts is challenging since WCM is a cryptic species complex. It includes multiple lineages that are distinguishable using mitochondrial (mtDNA COI, 16S) and nuclear (28S rDNA D2, ITS1–ITS2, and ANT) DNA sequences, differing also in host preference (Skoracka et al., 2012, 2013; Miller et al., 2013; Szydło et al., 2015). Some lineages are highly host-specific and locally distributed, whereas others are generalists with wider geographic ranges (Skoracka et al., 2014). Two WCM genotypes associated with wheat are the most polyphagous and widespread, having been found in the Middle East, Europe, Australia, and North and South America (Skoracka et al., 2014; Wosula et al., 2016). They are known as type 1 and type 2 in Australia (Carew et al., 2009) with corresponding genotypes occurring in North America (Hein et al., 2012), as well as in Europe and South America where they are known as MT-8 and MT-1, respectively (Skoracka et al., 2014). Hereafter this latter nomenclature will be used.

In North America these two lineages have been shown to transmit WSMV (Wosula et al., 2016). However, MT-1 had a higher reproductive capacity in the presence of WSMV and vectored it more efficiently than MT-8 (Seifers et al., 2002; Siriwetwiwat, 2006; Oliveira-Hofman et al., 2015). In Australia, among these two lineages only MT-1 has been observed to transmit WSMV (Schiffer et al., 2009). MT-1 is also the most effective vector of HPWMoV and TriMV (Seifers et al., 2002; McMechan et al., 2014; Wosula et al., 2016). Mixed-virus infections further confound virus–mite studies, e.g., transmission by MT-1 was more frequent from WSMV infected source plants than from those co-infected with TriMV (Oliveira-Hofman et al., 2015).

MT-8 and MT-1 have been found coexisting in mixed populations in wheat-producing areas in North America, Australia, and Europe, where plants from a single wheat field contained both MT-1 and MT-8 (Siriwetwiwat, 2006; Schiffer et al., 2009; Hein et al., 2012; Skoracka et al., 2017), further complicating management of viruses vectored by WCM. This sympatry combined with differential virus-transmission accentuates the need for efficient identification methods.

### WCM Management

To date, research to manage this mite–virus complex has focused mainly on the development of classical host plant resistance (HPR) to both the mite and viruses by introgressing favorable traits from resistant germplasm into advanced breeding lines (Whelan and Hart, 1988; Chen et al., 1998; Harvey et al., 2003; Malik et al., 2003a; Hakizimana et al., 2004; Carrera et al., 2012; Carver et al., 2016), in addition to cultural practices such as planting date and summer control of volunteer wheat plants (McMechan and Hein, 2016). The search for genes conferring WSMV resistance to wheat began shortly after the virus was identified in the 1950s (McKinney and Sando, 1951). With few sources of resistance available in wheat, the search eventually targeted close relatives culminating with the chromosome translocation of the *Wsm1* gene from *Thinopyrum intermedium* (Host) Barkworth & D.R. Dewey to the short arm of chromosome 4D in wheat (Friebe et al., 1991).

Continued efforts resulted in release of the first germplasm: KS96HW10-3 (Seifers et al., 1995) and first commercial cultivar ‘Mace’ (Graybosch et al., 2009) with the *Wsm1* gene. This gene has demonstrated resistance to both WSMV and TriMV (Friebe et al., 2009), however, its value has been limited due to linkage drag that reduces yields (Sharp et al., 2002). Similar issues have impacted a second gene, *Wsm3*, transferred into wheat from *T. intermedium* but efforts continue to improve its effectiveness and identify genetic markers (Friebe et al., 2009; Danilova et al., 2017).

A germplasm line, CO96093-2, was identified by Haley et al. (2002) as resistant to WSMV, but the gene’s origin was uncertain. Lu et al. (2011) found this gene to be a new gene (*Wsm2*) of wheat origin. Four varieties have thus far been released with the *Wsm2* gene: ‘RonL’ (Seifers et al., 2007), ‘Snowmass’ (Haley et al., 2011), ‘Clara CL’ (Martin et al., 2014), and ‘Oakley CL’ (Zhang et al., 2015). Studies with both *Wsm1* and *Wsm2* have demonstrated that both genes are temperature-sensitive with high levels of resistance below 20°C but breaking down as temperatures approach 25°C (Seifers et al., 1995, 2007).

Additional sources of WSMV resistance in wheat have recently been identified and hold promise for incorporation into commercial wheats (Seifers et al., 2007, 2013), including increased temperature stable resistance (Fahim et al., 2012a; Kumssa et al., 2017). Lu et al. (2011) has hypothesized the presence of a minor gene in wheat that confers partial resistance or tolerance in some commercial cultivars.

Early efforts to identify resistance to the WCM in wheat were not successful (Harvey and Livers, 1975), and this led to efforts to target close wheat relatives for resistance. Thus far, four WCM-resistance genes have been identified. The earliest of these genes (*Cmc3*) was translocated to wheat from rye (*Secale cereale* L.) (Martin et al., 1983; Malik et al., 2003a). It was present in ‘TAM 107,’ a commercial release that became widely used in the 1980s and 1990s across the Great Plains (Porter et al., 1987). However, the extensive use of TAM 107 led to loss of effectiveness of the gene (Harvey et al., 1995, 1997). A mite-resistance gene (*Cmc1*) translocated from *Aegilops tauschii* (Coss.) Schmal. to wheat (Thomas and Conner, 1986; Whelan and Thomas, 1989) has been used to develop breeding material (Cox et al., 1999) and the recent release of ‘Radiant’ in Canada (Thomas et al., 2012). A third source of resistance, *Thinopyrum ponticum* (Podp.) Barkworth & D.R. Dewey, contributed with gene *Cmc2* (Whelan and Hart, 1988). A second gene originating from *A. tauschii* (*Cmc4*) was found to be independent of *Cmc1* (Cox et al., 1999; Malik et al., 2003a) and has been used in the breeding release OK05312 (Carver et al., 2016). Additional resistance genes have been proposed but not yet isolated from common wheat (Harvey and Martin, 1992), rye (Cainong et al., 2010), and *A. tauschii* (Malik et al., 2003b; Dhakal et al., 2017).

The value of mite-resistance lies in the potential for reduced virus transmission and spread through the field, as well as in the reduction of mite buildup in the volunteer wheat that serves as a bridge host to the following wheat crop (Martin et al., 1984; Conner et al., 1991; Harvey et al., 2005). However, mite response to resistance genes has often been variable (Harvey et al., 1999) and the stability of resistance genes is a concern due to the apparent adaptation to *Cmc3* by mite populations (Harvey et al., 1995, 1997, 1999). Greater understanding of the variability in mite genotype responses to resistance genes is needed to evaluate potential stability of resistance genes. Genetic characterization of the mites used in resistance studies has become critical to understanding mite-gene response (Richardson et al., 2014; Aguirre-Rojas et al., 2017; Dhakal et al., 2017). Future efforts to pyramid *Wsm* and *Cmc* genes may enhance the utility and stability of these management options.

Molecular tools, such as *in situ* hybridization and genetic marker maps have improved the efficiency and precision of HPR introgression efforts. In addition, RNAi techniques have been used to produce transgenic wheat lines with resistance to WSMV (Fahim et al., 2010, 2012b; Cruz et al., 2014) and TriMV (Shoup Rupp et al., 2016) although no commercial wheat cultivars with this resistance have been released. With current advances in DNA sequencing technology, the whole genome sequences (WGSs) of wheat, WCM, WSMV, HPWMoV, TriMV, and BrSMV (Gustafson et al., 1987; Seifers et al., 2008; Stewart, 2016; Tatineni et al., 2016; Zimin et al., 2017) are all now available, presenting the opportunity to study these tripartite host–vector–virus relationships at the level of genome sequence and gene expression.

## Future Directions

### Wheat–WCM Interactions

Like many eriophyoid mites that attack grasses, WCM is vagrant, i.e., inhabiting the leaf surface rather than inducing galls, and there is very little published information regarding its direct molecular or physiological interactions with its hosts. Given the availability of its genome sequence and those of several of its hosts, such as wheat (Zimin et al., 2017), maize (Schnable et al., 2009), and barley (Mascher et al., 2017), WCM is a good candidate to be a model for such studies in grass-infesting Eriophyoidea. For example, using available genomic and transcriptomic (Ozsolak and Milos, 2011; Jänes et al., 2015) resources, it will be possible to determine whether the ability of polyphagous genotypes (e.g., MT-1, MT-8) to change from one host to another is genetically or epigenetically (Laird, 2011) controlled. Similarly, the factors that determine which plant species are accepted by a host-specific WCM genotype can be dissected (Gompert et al., 2010; Narum et al., 2013). Moreover, novel genomic technologies and high-throughput phenotyping of wheat varieties can accelerate germplasm improvement (see Mondal et al., 2016 for examples).

Proteomic analyses of rice leaves from control plants and those infested with *Schizotetranychus oryzae* (Acari: Tetranychidae) revealed a wide range of intracellular physiological changes induced by this mite although the specific source(s) of induction (e.g., salivary components) are not known (Buffon et al., 2016). Similar analyses of WCM on one or more of its hosts could take advantage of the mite’s and host plants’ genomic resources, as well as recent techniques developed to characterize the salivary proteins of a tetranychid mite (Jonckheere et al., 2016), to assess mite–host interactions from both sides. Effects of individual proteins could be assessed through knockout genotypes created by the CRISPR-Cas9 mutagenesis (Ran et al., 2013). Complementary studies of other eriophyoids and mite species from other families that attack cereal crops would identify similarities and differences in these interactions that could shed light on prospective control strategies against multiple mite species, e.g., via RNAi in the host plant to block production of essential mite proteins.

### WCM–Virus Interactions

Regarding the ability of mites to transmit WSMV, a genotyping-by-sequencing study (e.g., Narum et al., 2013) incorporating all known WCM genotypes with variable WSMV transmission ability and anchored to an annotated WGS of WCM would identify candidate genomic regions associated with WSMV transmission variability. This could also be used to explore the differential transmission of TriMV and HPWMoV by WCM genotypes. Complementary transcriptomic and epigenetic studies could further identify the candidate gene(s) involved in this variability and tease apart genetic and epigenetic factors.

Different strains of WSMV have also been detected that are differentially transmitted by individual WCM genotypes (Wosula et al., 2016). Mutations to the helper component proteinase (HC-Pro) gene of WSMV have been shown to alter transmission from mite to plant or prevent it altogether (Stenger et al., 2006; Young et al., 2007) although the precise physiological mechanism of transmission is unknown. Given that WSMV is a circulative virus that is transmitted via the salivary glands of WCM (Paliwal, 1980), the development of salivary protein characterization techniques (Jonckheere et al., 2016) may enable association of specific WCM salivary proteins with successful or unsuccessful WSMV transmission. If other WCM-transmitted viruses have a persistent circulative type of relationship with the vector, understanding the mechanisms (receptors) by which these viruses cross the midgut epithelium and salivary gland barriers to reach the stylet channel may yield basic information regarding the traffic of these viruses within the mite body.

### WCM Colonization Potential

The spread of WCM and its associated plant viruses to cereal-producing regions worldwide is of increasing scientific and economic importance (Navia et al., 2013). Colonization and invasive potential of any organism is inevitably associated with its dispersal ability and its degree of ecological specialization (Ehrlich, 1986). WCM disperses passively by air currents (Sabelis and Bruin, 1996) and wheat-associated lineages are characterized by low host-specificity (Skoracka et al., 2013). Generalists with high dispersal ability are typically successful invaders (Wilson et al., 2009). But relationships between WCM dispersal potential, degree of host specialization, and colonization success have never been tested. To do so, it will be necessary to understand the mechanisms of successful WCM wheat colonization, including long-established and recent invasions. Research on the relationship between WCM host specialization and dispersal ability revealed trade-offs in plant performance between different host plant species after mite dispersal (Laska et al., 2017). Also it has been shown that a small number of WCM specimens landing on wheat plants after aerial dispersal (about 2% of an initial source population) were able to establish dense colonies very quickly, indicating great colonization potential (Kiedrowicz et al., 2017). Understanding how interactions between dispersal and local adaptation shape WCM distribution is crucial because predicting spread of potentially invasive organisms, particularly under current anthropogenic environmental changes, is a key to managing pest outbreaks and minimizing ecosystem degradation.

## Author Contributions

AS, BR, and GH designed the conception, wrote the manuscript, and read and approved the submitted version with equal contribution.

## Conflict of Interest Statement

The authors declare that the research was conducted in the absence of any commercial or financial relationships that could be construed as a potential conflict of interest.
